# Ipilimumab treatment results in an early decrease in the frequency of circulating granulocytic myeloid derived suppressor cells as well as their arginase1 production

**DOI:** 10.1186/1479-5876-12-S1-O9

**Published:** 2014-05-06

**Authors:** Giusy Gentilcore, Coaña Pico de Yago, Isabel Poschke, Yumeng Mao, Maria Nyström, Johan Hansson, Giuseppe V Masucci, Rolf Kiessling

**Affiliations:** 1 Department of Oncology and Pathology, Cancer Center Karolinska, Karolinska Institutet, Stockholm, Sweden; 2 Division of Molecular Oncology of Gastrointestinal Tumors, German Cancer Research Center, Heidelberg, Germany

## Background

Ipilimumab (Yervoy) is a fully human antibody that blocks CTLA-4 and has proven to extend overall survival in patients with unresectable stage III or stage IV melanoma [1]. Immune related adverse effects (IRAE) are frequent and can be severe but are reversible with early diagnosis and can be managed with corticosteroid therapy [2]. Most of the recently published immune monitoring studies focus mainly on the effect that ipilimumab has on T cell populations [3]. To date, little information is available on the possible impact that ipilimumab treatment may have on MDSC populations and their suppressive mechanisms [4]. In order to evaluate these effects, we conducted an in-depth immune monitoring study centered on peripheral blood MDSC populations as well as T cells in advanced melanoma patients undergoing treatment with ipilimumab.

## Materials and methods

Fourteen patients with advanced stage melanoma received ipilimumab treatment at 3 mg/kg or 10 mg/kg doses and six of which were part of an ongoing double blind randomized trial (Bristol-Myers Squibb trial CA184-169). Blood samples were collected from each patient before treatment (baseline) and at the time of the second and fourth ipilimumab doses (3 and 9 weeks after the first dose). Peripheral blood mononuclear cells were isolated by density gradient centrifugation and stained for multicolored flow cytometric analysis. The staining protocol included five 9-color panels to analyze T cells (relative frequencies, activation, memory and Tregs) and MDSCs.

## Results

Absolute lymphocyte counts showed an increasing trend during the course of treatment without significant differences. Analysis of circulating Treg (CD4+ CD25+ CD127lo FoxP3+) frequencies revealed an initial increase, significantly decreasing after the second ipilimumab dose (week 9). The endpoint mean frequency of Tregs was lower than the baseline. Changes in MDSC populations with granulocytic and monocytic phenotype (Lin- HLA-DR-/lo CD15+ CD33+ CD11b+ and Lin- HLA-DR-/lo CD14+, respectively) were monitored and the results showed that, after the first dose, the granulocytic MDSC population significantly decreased, remaining low at week 9. This decrease was accompanied by a significant decrease in the population of ARG1+ myeloid cells.

## Conclusion

The results shown in this work provide a first look at the early responses of peripheral blood myeloid cell populations to ipilimumab treatment. The mechanisms by which these *in trans* effects are taking place should be further explored as well as their possible relations to clinical benefit.
